# Validity of EQ-5D-5L breathing and cognition bolt-ons in non-hospitalized patients after COVID-19

**DOI:** 10.1007/s11136-025-04133-4

**Published:** 2026-01-09

**Authors:** Knut Stavem, Andrew M. Garratt

**Affiliations:** 1 Health Services Research Unit, Akershus University Hospital, Lørenskog, Norway; 2 Institute of Clinical Medicine, University of Oslo, Campus Ahus, Lørenskog, Norway; 3 Department of Pulmonary Medicine, Medical Division, Akershus University Hospital, Lørenskog, Norway; 4 Division for Health Services, Norwegian Institute of Public Health, Oslo, Norway

**Keywords:** EQ-5D-5L, Quality of life, Bolt-on, Breathlessness, Cognition, Validity

## Abstract

**Purpose:**

The EQ-5D has been criticized for lacking dimensions for breathing and cognition. In acute COVID-19 and Long COVID, breathing problems and cognitive complaints are common. This study evaluated the inclusion of EQ-5D-5L (bolt-on) items that assess these dimensions.

**Methods:**

In a follow-up of community-based non-hospitalized patients with COVID-19 in 2020 (n = 450), we used respiratory and cognitive bolt-on items alongside other questionnaires about 30 months later (n = 220). We assessed data quality and construct validity for the bolt-ons, including comparison with concurrently used instruments’ domain and item scores. Bolt-on scores were correlated with pulmonary function tests and tablet-based cognitive tests about 1 year earlier. Finally, we assessed the contribution of the bolt-ons to variability of the EQ VAS.

**Results:**

Most patients had none or slight problems with breathing and cognition. The breathing problems bolt-on had an 8% lower ceiling effect, but otherwise a similar response distribution to the mMRC. Cognition bolt-on and four DSQ-SF item scores relating to frequency and severity were similar, except 2–13% higher ceiling effects for the former. The breathing bolt-on had a rank correlation of 0.54 with mMRC scores and higher correlations with Dyspnoea-12 scale scores. EQ-5D-5L dimensions explained 54% of variation in EQ VAS scores; the breathing bolt-on contributed a further 2%, but the cognition bolt-on contributed very little.

**Conclusion:**

The results support the validity of the two bolt-ons in follow-up of non-hospitalized patients after COVID-19. Adding items of respiratory and cognitive symptoms may enhance the EQ-5D's appropriateness in respiratory and cognitive research and clinical practice.

## Introduction

The EQ-5D-5L descriptive system comprises five dimensions (mobility, self-care, usual activities, pain/discomfort, and anxiety/depression), each with five levels. The brevity of the questionnaire offers important advantages including low respondent burden, and the instrument has widespread application in research and quality measurement. However, the descriptive system has been criticized for not including important dimensions of health, which has implications for content validity and measurement properties including construct validity and responsiveness to change in specific health problems.

Additional dimensions, referred to as ‘bolt-ons’, have been developed for use alongside the EQ-5D [1], and a systematic review identified 26 examples covering several dimensions of health [2]. Bolt-ons, such as breathing, cognition, and fatigue, may improve content validity and psychometric properties through the addition of dimensions of relevance to specific settings and health problems. Breathlessness is a widespread complaint in the general population and increases with age [3, 4]. There are various methods available for assessing the prevalence of breathlessness, which varies considerably according to population [5]. The most common self-completed method is use of the modified Medical Research Council (mMRC) questionnaire, a 5-point ordinal scale [6]. More comprehensive instruments include the Dyspnea-12 [7–9]. The EQ-5D is often used in respiratory diseases, such as asthma and chronic obstructive pulmonary disease (COPD) [10, 11], but EQ-5D dimensions omit important aspects of these diseases. Correlations of EQ-5D and other generic health-related quality of life (HRQoL) instrument scores with lung function and respiratory markers are usually low or moderate. EQ-5D respiratory bolt-ons that are designed to improve responsiveness in such diseases, were recently developed and tested in patients with chronic obstructive pulmonary disease (COPD) [12, 13].

Cognitive functioning and concentration are regarded as other potentially important bolts-on dimensions to the EQ-5D-5L [1, 14–16]. A cognitive bolt-on was recently developed and tested in patients after trauma, stroke and Q-fever [16–18]. Respiratory and cognitive symptoms, such as brain fog, are prominent both in acute COVID-19 and in Long COVID [19–22]. Long COVID can be defined several ways including an interim federal working definition from the National Research Action Plan on Long COVID [23]: ‘Long COVID is broadly defined as signs, symptoms, and conditions that continue or develop after initial COVID-19 or SARS-CoV-2 infection. The signs, symptoms, and conditions are present 4 weeks or more after the initial phase of infection; may be multisystemic; and may present with a relapsing–remitting pattern and progression or worsening over time, with the possibility of severe and life-threatening events even months or years after infection. Long COVID is not one condition. It represents many potentially overlapping entities, likely with different biological causes and different sets of risk factors and outcomes.’ In contrast, other definitions require persistent symptoms for 8 or 12 weeks, and some allow for fluctuation of symptoms [24]. Long COVID affects 10–50% of COVID-19 survivors [25, 26], though mechanisms for the condition are largely unknown. However, emerging evidence suggests a dissociation between pathophysiology and symptom burden, particularly in non-severe COVID-19 where initial cardiopulmonary damage may be trivial [27].

This study aimed to evaluate two EQ-5D-5L bolt-ons in a cohort of non-hospitalized patients with long-term follow-up after a positive PCR-test for COVID-19 in 2020. The specific study aims were to:

1. Assess the construct validity of the breathing problems and cognitive functioning bolt-ons in patients about 30 months after acute COVID-19
2. Compare bolt-on results with those from disease-specific instruments, and
3. Determine the contribution of the two bolt-ons to explaining the variability in HRQoL, as assessed with the EQ VAS.

## Methods

This was a secondary analysis of data from a follow-up study of non-hospitalized patients after COVID-19 in 2020 in Norway (n = 450).

### Study population

This study comprises a geographical cohort in the catchment areas of two Norwegian hospitals, Akershus University Hospital and Østfold Hospital, with a combined population of about 900,000 inhabitants, i.e., about 17% of the population of Norway. We initially invited subjects ≥ 18 years with a positive polymerase chain reaction (PCR) test for severe acute respiratory syndrome coronavirus-2 (SARS-CoV-2) before June 1, 2020 which came from the hospital microbiology laboratories and the largest private microbiology laboratory in the area, Fürst Medical Laboratory. We anticipated that these labs would have analyzed > 90% of the SARS-CoV-2 tests in this area. Patients were eligible if they had not been admitted to hospital < 22 days after the positive PCR test. We excluded patients that lived outside the hospitals’ catchment areas, had no valid 11-digit Norwegian national identity number, permanent address in a nursing home, or dementia [28, 29].

Following exclusions, we invited 938 subjects to a survey, and 458 responded after one postal reminder [28–31]. Between December 2020 and April 2021, we invited respondents to the first survey, while excluding those declining further participation (n = 19), to a follow-up visit including blood samples, further questionnaires, pulmonary function tests and tablet-based cognitive testing.

**Fig. 1.**
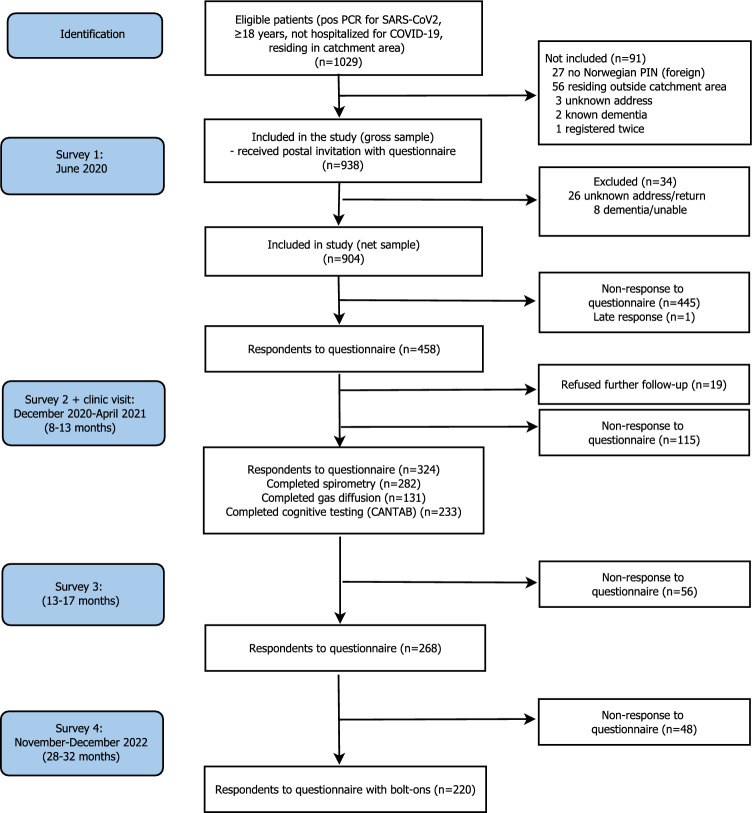
Flow of patients through study

Participants received follow-up questionnaires after 13–17 months (3rd round) and 28–32 months (4th round; November/December 2022). The present study used information from the 4th round (n = 220 respondents), in addition to information from the baseline survey and the clinic visit during the 3rd round including testing of pulmonary (n = 280) and cognitive function (n = 233) (Fig. 1).

### EQ-5D-5L bolt-ons

Breathing and cognition bolt-ons were included alongside the EQ-5D-5L questionnaire in the 4th round of the COVID-19 study, in November/December 2022.

#### Respiratory

Two respiratory bolt-on dimensions were recently developed to improve the responsiveness of EQ-5D in respiratory diseases, such as chronic obstructive pulmonary disease (COPD) and asthma [12], after review of existing disease-specific HRQoL instruments and statistical analysis of empirical data. Their importance was assessed in a pilot study, determining their usefulness relative to the five core EQ-5D dimensions. It was concluded that a breathing problems item was the most appropriate respiratory bolt-on and provided the greatest utility decrements [12]; however, this gave only small improvements in EQ-5D-5L performance in another study [13].

We used this respiratory bolt-on (beta version) [12], which we adapted for use in Norwegian, based on available English version, and harmonization with an existing Swedish translation (Appendix 1).

#### Cognition

The cognitive functioning bolt-on was originally added to the 3L version to broaden the scope of the EQ-5D [14]. It was further adapted to the EQ-5D-5L descriptive system, using the same response categories (EQ-5D-5L + Cognition)[18]. This bolt-on dimension was recently used in patients after trauma [16], and after Q-fever [17], where long-term problems with cognition is a common challenge. The latter study reported that adding the cognition dimension to the five existing EQ-5D dimensions had almost no impact on the explained variance. However, to date few studies have evaluated this bolt-on. The cognition bolt-on was also adapted from the English version and harmonized with a Swedish version (Appendix 2).

### Other self-rated instruments/items in the 4th round

#### Respiratory questionnaires

- The modified Medical Research Council Questionnaire (mMRC) dyspnea scale [6] assesses perceived breathlessness at different grades of strenuous activities on a 5-point scale (0 = no breathlessness except on strenuous exercise; 1 = shortness of breath when hurrying on the level or walking up a slight hill; 2 = walks slower than people of same age on the level because of breathlessness or has to stop to catch breath when walking at their own pace on the level; 3 = stops for breath after walking ∼100 m or after few minutes on the level; and 4 = too breathless to leave the house, or breathless when dressing or undressing) [32]. mMRC scores ≥ 2 correspond with breathlessness of clinical importance [4].
- Dyspnoea-12 (D-12) comprises 12 items on a 4-point scale that give two scales: physical (range 0–21) and emotional (range 0–15), and a total scale (range 0–36) with higher scores indicating more symptoms [7]. It has evidence for validity in patients with asthma and COPD, including Norwegian patients with COPD [33].

#### Cognition items

- The short-form DePaul Symptom Questionnaire (DSQ-SF) [34] includes four items phrased as frequency and severity of problems with memory (‘problems remembering things’) or trouble keeping attention for long (‘difficulty paying attention for a long period of time’) (over the past 6 months). Items use 5-point scales of frequency (0 = none of the time, 1 = a little of the time, 2 = about half of the time, 3 = most of the time, and 4 = all of the time) and severity (0 = symptom not present, 1 = mild, 2 = moderate, 3 = severe, 4 = very severe).
- The Chalder Fatigue Questionnaire (CFQ-11) includes four items assessing aspects of cognition (do you have difficulty concentrating, do you make slips of the tongue when speaking, do you find it more difficulty to find the right word, how is your memory) using 4-point scales (0 = less than usual, 1 = no more than usual, 2 = more than usual, 3 = much more than usual) summed to a mental fatigue subscale from 0–12 [35, 36].

#### Symptoms of acute COVID-19 and comorbidity

- Symptoms during the acute phase of COVID-19 were assessed retrospectively at 3 months using a checklist of 23 self-reported symptoms, largely adopted from the WHO/ISARIC platform. The severity of the acute COVID-19 phase will be quantified using the number of these retrospectively reported symptoms. In addition, we used the single items of ‘Breathlessness’ (yes/no) and ‘Confusion’ (yes/no) during acute COVID-19.
- Comorbidity was recorded using a checklist of 21 diseases and conditions, 18 of which constitute a self-report version of the Charlson comorbidity index [37].

#### Objective tests

In a previous round of the study, about 8–13 months after COVID-19, we collected:

- Data on lung function (spirometry, carbon monoxide gas diffusion) (n = 280) using standard procedures [38, 39].
- A comprehensive tablet-based cognitive test, Cambridge Neuropsychological Test Automated Battery (CANTAB), comprising four tests for short-term memory, attention and executive function (n = 233) [28, 40]. The participants responded to one warm-up task, a motor screening test (MOT), and four tests: (1) Delayed matching to sample (DMS), testing short term memory, visuospatial processing, learning and attention; (2) One-touch Stockings of Cambridge (OTS), testing executive function, which includes high level thinking and decision-making processes such as mental flexibility, planning and problem solving; (3) Rapid visual information processing (RVP), testing sustained attention; (4) Spatial working memory (SWM), testing working memory and strategy. The whole battery was expected to take 34 min to complete. The four tests were administered in the following order: RVP with three targets (9 min), DMS (7 min), OTS (10 min), and the SWM (6 min). The tests were available in Norwegian and English and had UK population norms from the vendor for subjects aged 18–85 + years, except for the MOT. The CANTAB scores are adjusted for age, sex and education and presented as z-scores for comparison the population norms, i.e. number of SDs below the reference population mean of 0. Reference values are not available for Norway.

### Statistical analysis

We present descriptive statistics and compare the performance of the bolt-ons with existing assessment tools (mMRC, D-12 and the cognition items from the DSQ-SF and CFQ-11). We assessed levels of missing data and ceiling effects of the bolt-ons, the latter according to the proportion of patients reporting ‘no problems’, and compared to the EQ-5D-5L, the mMRC, and cognition items from the DSQ-SF and CFQ-11 at about 30 months.

We used data from the other instruments/items at 30 months and from spirometry and cognitive testing at 8–13 months to assess the construct validity of the bolt-ons by hypothesis testing corresponding to recommended criteria [41]. Spearman’s rank correlation coefficient (ρ) was used in hypothesis testing:

#### Breathing bolt-on

- We hypothesised that the bolt-on scores would have correlations ≥ 0.6 with those measuring the same construct [42]: D-12 scale and mMRC scores. Whilst assessing the same construct, breathlessness during acute COVID-19 was assessed much earlier at 3 months and hence a much lower level of correlation was expected. Given the content of the bolt-on, these correlations were expected to be higher than the correlations with responses to the five EQ-5D-5L dimensions.
- We assessed the correlations with commonly used markers of respiratory disease (spirometry variables, transfer factor for carbon monoxide of the lung [TLCO] at 8 months, i.e., about 20 months earlier). Lower correlations of 0.20–0.50 were expected for bolt-on scores with these clinical measurements. Given the content of the bolt-on, we hypothesized that these would be higher than those for the EQ-5D-5L dimension scores.
- We also hypothesised that bolt-on responses would be have correlations of 0.20–0.50 with the number of symptoms during acute COVID-19, and their associations were expected to be higher than for the other EQ-5D-5L dimensions.

#### Cognition bolt-on

- We hypothesised that the bolt-on scores would have correlations ≥ 0.60 with those measuring the same construct [42]: DSQ-SF items, CFQ-11 items and mental fatigue subscale. Given the content of the bolt-on, these correlations were expected to be higher than the correlations with responses to the five EQ-5D-5L dimensions. Lower correlations, 0.20–0.50, were expected for the CANTAB scores at 8–13 months after acute COVID-19, but higher than for the other EQ-5D-5L dimensions.

We finally determined the contribution of adding the bolt-ons to the five EQ-5D-5L items in multiple linear regression analysis with the EQ VAS as the dependent variable. If the bolt-ons were making an important contribution as measures of health in this patient population, they should explain additional variation in EQ VAS scores compared to the five EQ-5D-5L dimensions. We used Stata version 18.0 (Stata Corp, College Station, TX) for all analyses.

## Results

In total, 220 participants responded to the survey at about 30 months after acute COVID-19, median (25th to 75th percentile) 959 (949 to 968.5) days after the positive PCR test for SARS-Cov2) and were included in the analysis. The respondents had a mean (SD) age of 53.2 (14.3) years, and 133 (60%) were females. For further descriptive statistics for the respondents and their condition during acute COVID-19, see Table 1.

**Table 1:** Descriptive Statistics for respondents during COVID-19. Number (%) unless otherwise stated

N	220
Age in years, mean (SD)	53.2 (14.3)
Sex	
Female	133 (60)
Male	87 (40)
Norwegian origin	
No	18 (8)
Yes	201 (92)
Body mass index, self-report kg/m^2^ (*n* = *217*), mean (SD)	27.0 (5.4)
No. of comorbidities (0–21 scale), median (range)	1 (0–6)
No. of comorbidities, categorized	
0	102 (46)
1	71 (32)
2	24 (11)
≥ 3	23 (11)
Highest attained educational level	
Primary school (< 11 years)	16 (7)
Secondary school (11–13 years)	80 (36)
University (> 13 years)	124 (56)
No. of COVID-19 symptoms (0–23 scale), median (range)	9 (1–18)
No. of COVID-19 symptoms, 3 categories	
0–5	54 (24)
6–9	83 (38)
10–23	83 (38)
Confusion during acute COVID-19	
No	188 (85)
Yes	32 (15)
Dyspnea during acute COVID-19	
No	95 (43)
Yes	125 (57)

### Questionnaire and objective test results

The distribution of item scores across instruments after 30 months is shown in Table 2. For the breathing bolt-on, 54% reported no problem, 40% slight problem, 6% moderate problems, and under 1% reported severe problems with breathing. No respondent reported extreme problems (Table 2). The distribution of scores on the mMRC scale was very similar, with 66% scoring in lowest category (class 0), 26% in class 1, and none scoring highest (class 4). In total, 6% reported moderate to extreme problems on the breathing bolt-on and 8% reported corresponding mMRC scores ≥ 2.

**Table 2:** Distribution of questionnaire item scores at about 30 months (N = 220). Number (%)

Domain	Instrument	Response categories				
	*EQ-5D-5L*	1 No problem	2 Slight	3 Moderate	4 Severe	5 Extreme problem
Health status	Mobility	175 (80)	34 (16)	8 (3.6)	2 (0.9)	1 (0.5)
	Self-care	205 (93)	13 (5.9)	1 (0.5)	1 (0.5)	0 (0)
	Usual activities	141 (64)	63 (29)	13 (5.9)	3 (1.4)	0 (0)
	Pain/discomfort	64 (29)	103 (47)	39 (18)	13 (5.9)	1 (0.5)
	Anxiety/depression	131 (60)	67 (31)	18 (8.2)	3 (1.4)	1 (0.5)
	*Bolt-ons*					
	Breathing	119 (54)	88 (40)	12 (5.5)	1 (0.5)	0 (0.0)
	Cognition	88 (40)	104 (47)	21 (9.5)	6 (2.7)	1 (0.5)
		0 Only breathless with strenuous exercise	1 Breathless when hurrying on the level or walking up a slight hill	2 Walk slower than people the same age because of breathlessness, or stopping for breath when walking on own pace on the level	3 Stop for breath after walking about 100 m or after a few minutes on the level	4 Too breathless to leave the house or breathless when dressing or undressing
Dyspnea	*modified Medical Research Council dyspnea scale*	143 (66)	57 (26)	16 (7)	2 (1)	0 (0)
Cognition	*CFQ-11 mental*	0 Less than usual	1 No more than usual	2 More than usual	3 Much more than usual	
	8. Do you have difficulty concentrating?	6 (3)	131 (60)	68 (31)	15 (7)	
	9. Do you make slips of the tongue when speaking?	5 (2)	176 (80)	32 (15)	7 (3)	
	10. Do you find it more difficult to find the right word?	4 (2)	120 (55)	80 (36)	16 (7)	
	11. How is your memory?	1 (0.5)	120 (55)	89 (40)	10 (5)	
	*DSQ-SF neurocognitive items -frequency*	0 None of the time	1 A little of the time	2 About half of the time	3 Most of the time	4 All of the time
	Problems remembering things	59 (27)	120 (55)	27 (12)	10 (5)	4 (2)
	Difficulty paying attention for a long period of time	73 (33)	105 (48)	28 (13)	11 (5)	3 (1)
	*DSQ-SF neurocognitive items—severity*	0 Symptom not present	1 Mild	2 Moderate	3 Severe	4 Very severe
	Problems remembering things*	63 (29)	95 (43)	41 (19)	15 (7)	5 (2)
	Difficulty paying attention for a long period of time**	82 (38)	86 (39)	30 (14)	16 (7)	4 (2)

DSQ-SF = De Paul Symptom Questionnaire, short form

CFQ-11 = Chalder Fatigue Questionnaire, 11 items

^*^n = 219, ^**^n = 218

For the cognition bolt-on, 192 (87%) reported no or slight problems which was similar to DSQ-SF frequency item scores, where 179 (82%) reported problems remembering things and 178 (81%) reporting difficulty paying attention for a long time none/a little of the time. For the DSQ-SF severity items, 72% reported that problems remembering things was not present/mild, while 76% reported the same for difficulty paying attention for a long time. Across the four CFG-11 mental fatigue items, 55–80% reported that cognitive difficulties were ‘no more than usual’.

The respondents had a mean EQ-5D index score of 0.788 (SD 0.169) and EQ VAS score of 72.7 (18.0) (Table 3). Spirometry values were slightly below the population averages, as indicated by the small negative z-scores for FVC and FEV_1_, which are reported alongside other lung function variables and the tablet-based cognitive tests (Table 4). For the four cognitive tests (n = 137), the mean scores ranged from 0.366 SD below to 0.441 SD above the mean of the UK reference population.

**Table 3:** Scores on composite scales and visual analog scale for dyspnea, mental fatigue/cognition and general health at 30 months after COVID-19 (n = 220). Higher score is worse health/more symptoms

	No. of items	Range	Mean (SD)
Dyspnea-12 physical	7	0–21	2.0 (3.2)
Dyspnea-12 affective	5	0–15	1.0 (2.2)
Dyspnea-12 total	12	0–36	2.9 (5.1)
CFQ-11 mental	4	0–12	5.6 (2.0)
EQ-5D index (crosswalk)*	5	−0.594–1.00	0.788 (0.169)
EQ VAS*	1	0–100	72.7 (18.0)

CFQ-11 = Chalder fatigue scale (11-item version)

^*^Higher score represents better health

**Table 4:** Lung function tests and tablet-based cognitive tests at 8–13 months after acute COVID-19

	n	Mean (SD)
*Lung function variables*		
FVC, L	163	4.0 (1.0)
FEV_1_, L	163	3.0 (0.8)
FEV_1_/FVC	163	0.75 (0.07)
TLCO, mmol/(min*kPa)	70	8.0 (2.2)
*Lung function variables, z-scores*		
FVC	163	−0.170 (0.929)
FEV_1_	163	−0.475 (0.969)
FEV_1_/FVC	163	−0.589 (0.795)
TLCO	70	0.023 (1.321)
6 min walk distance, m	163	600 (117)
*Cognitive tests (CANTAB), z-scores*		
Delayed matching to sample (DMS)	137	−0.366 (1.152)
One-touch stockings of Cambridge (OTS)	137	0.108 (1.022)
Rapid visual information processing, three targets (RVP)	137	0.441 (1.117)
Spatial working memory (SWM)	137	−0.183 (1.193)

FVC: Forced vital capacity

FEV_1_: Forced expiratory volume in 1 s

TLCO: Transfer factor of the lung for carbon monoxide

### Assessment of construct validity

#### Breathing bolt-on

The correlation between the breathing bolt-on item and mMRC scores was 0.539, and ranged 0.611–0.736 with the D-12 scale scores. These were all higher than those for the five EQ-5D-5L dimensions (range 0.166–0.479); usual activities (range 0.409–0.479) and pain/discomfort (range 0.364–0.445) had the highest correlations with the mMRC and D-12 scales, and self-care (range 0.166–0.219) and anxiety/depression (range 0.154–0.275) had the lowest (Table 5). Correlations between the breathing bolt-on scores and number of symptoms or having dyspnea during acute COVID-19 were low, but of a similar level as those for mMRC scores.

**Table 5:** Spearman rank correlations (rho) of the Breathing bolt-on and modified Medical Research Council dyspnea scale (mMRC) with other measurements at 30 months

Questionnaires/items	No. of items	n	Breathing bolt-on	mMRC	Mobility	Self-care	Usual activities	Pain/ discomfort	Anxiety/ depression
*Breathing bolt-on*	1	220			0.272	0.186	0.394	0.366	0.162
*mMRC*	1	218	0.539		0.448	0.166	0.479	0.364	0.154
*Dyspnea-12 scales*									
Total	12	220	0.736	0.586***	0.351	0.216	0.478	0.445	0.252
Physical subscale	7	220	0.721	0.558***	0.342	0.219	0.470	0.437	0.239
Affective subscale	5	220	0.611	0.52***	0.312	0.184	0.409	0.411	0.275
*No. of symptoms during acute COVID-19* (0–23 scale)*	23	220	0.257	0.230***	0.167	0.105	0.335	0.336	0.297
Dyspnea during acute COVID-19*	1	220	0.227	0.244***	0.156	0.092	0.164	0.154	0.164
Measurements**									
FEV_1_, L		163	−0.298	−0.315****	−0.215	−0.151	−0.245	−0.32	−0.152
FVC, L		163	−0.298	−0.347****	−0.175	−0.159	-0.282	−0.319	−0.128
FEV_1_/FVC		163	−0.053	−0.036****	−0.224	−0.011	0.031	−0.097	−0.111
TLCO, mmol/(min*kPa)		70	−0.141	0.0013	−0.115	−0.2	-0.188	−0.234	−0.002
6 min walk distance, m		163	−0.306	−0.313****	−0.284	−0.169	-0.323	−0.407	−0.273

FEV_1_: Forced expiratory volume in 1 s

FVC: Forced vital capacity

TLCO: Transfer factor of the lung for carbon monoxide

^*^Reported retrospectively at 3 months

^**^At 8–13 months after acute COVID-19

^***^n = 218

^****^n = 162

The breathing bolt-on item showed a similar pattern of correlations with the D-12 scales, although with slightly higher values, than for the mMRC dyspnea scale. In contrast, correlations for the breathing bolt-on were lower with two of the other EQ-5D-5L items (mobility, usual activities) than for the mMRC dyspnea scale.

Correlations for breathing bolt-on scores and prior lung function tests were low to moderate and similar to those for the mMRC. Comparable correlations for the EQ-5D-5L dimension scores of usual activities and pain/discomfort were slightly higher in some cases; both compared to the bolt-on and mMRC scores.

#### Cognition bolt-on

The cognition bolt-on item showed high correlations with the four DSQ-SF neurocognitive items (range 0.681–0.743), which were substantially higher than those for the EQ-5D-5L dimensions (range 0.189–0.489) (Table 6). The cognition bolt-on item also had largely moderate to high correlations with the four CFQ-11 items (range 0.375–0.719) and the mental fatigue scale (0.746) that all were considerably higher than comparable correlations for the EQ-5D-5L dimensions. Correlations were low between self-reported confusion during acute COVID-19 and the cognition bolt-on or the other EQ-5D-5L items scored at follow-up after 30 months.

**Table 6:** Spearman rank correlations (rho) of the Cognition bolt-on with other item and scale scores 30 months

Questionnaires/items	No. of items	n	Cognition bolt-on	Mobility	Self-care	Usual activities	Pain/ discomfort	Anxiety/ depression
*Cognition bolt-on*	1	220		0.225	0.189	0.423	0.278	0.235
*De Paul symptom questionnaire (DSQ-SF) neurocognitive items*								
Problems remembering things, frequency	1	220	0.742	0.192	0.201	0.419	0.294	0.262
Difficulty paying attention for a long period of time, frequency	1	220	0.681	0.272	0.277	0.489	0.339	0.252
Problems remembering things, severity	1	219	0.743	0.203	0.194	0.417	0.337	0.262
Difficulty paying attention for a long period of time, severity	1	218	0.687	0.219	0.230	0.455	0.343	0.308
*Chalder fatigue questionnaire(CFQ-11) cognition items*								
Mental fatigue subscale	4	220	0.746	0.235	0.214	0.483	0.333	0.314
Do you have difficulty concentrating?	1	220	0.559	0.247	0.147	0.452	0.294	0.320
Do you make slips of the tongue when speaking?	1	220	0.375	0.114	0.254	0.297	0.221	0.265
Do you find it more difficult to find the right word?	1	220	0.639	0.143	0.224	0.391	0.284	0.175
How is your memory?	1	220	0.719	0.202	0.151	0.386	0.266	0.271
Confusion during COVID-19*	1	220	0.197	0.188	0.094	0.145	0.129	0.135
CANTAB test battery, z-scores**								
Delayed matching to sample (DMS)		137	0.082	-0.125	0.049	-0.037	-0.028	0.170
One-touch stockings of Cambridge (OTS)		137	0.01	-0.057	0.013	0.045	-0.099	-0.031
Rapid visual information processing, three targets (RVP)		137	0.242	0.083	-0.064	0.164	-0.036	0.039
Spatial working memory (SWM)		137	0.065	-0.063	-0.029	-0.141	-0.100	-0.240

^*^Reported retrospectively at 3 months

^**^At 8–13 months after acute COVID-19

The correlations between the cognition bolt-on item scores and objective tablet-based cognitive tests about 20 months earlier were low and varied from low negative to low positive values. The highest of these correlations was between the cognition bolt-on and the RVP test of the CANTAB battery (ρ = 0.242).

### Contribution to variability of the EQ VAS and ceiling effects

In multiple linear regression analysis with EQ VAS as the dependent variable, a model with the five standard EQ-5D-5L items had an R^2^ of 0.542 (Table 7). When adding the breathing bolt-on to the model, the R^2^ increased to 0.565. Adding the cognition bolt-on did not contribute to increasing the R^2^ in either of these two models.

**Table 7:** Explanatory power of the five E-5D-5L dimensions and added bolt-ons to explain variability in the EQ VAS at 30 months after COVID-19, adjusted R^2^ from multiple linear regression models (n = 220), and percentage scoring the best possible health (ceiling effect)

	Model 1	Model 2	Model 3	Model 4
No. of items	5	6	6	7
*EQ-5D-5L Items*				
Mobility	x	x	x	x
Usual activities	x	x	x	x
Self-care	x	x	x	x
Pain/discomfort	x	x	x	x
Anxiety/depression	x	x	x	x
*Bolt-on items*				
Breathing		x		x
Cognition			x	x
Adjusted R^2^	0.542	0.565	0.543	0.561
No. scoring 1 for all items (at ceiling)	47/220 (21.4%)	35/220 (15.9%)	31/220 (14.1%)	24/220 (10.9%)

For five dimensions of the EQ-5D-5L, 21.4% of respondents scored at the ceiling or highest possible level equivalent to 1 for the EQ-5D index (Table 7). Following inclusion of the breathing bolt-on as a 6th item in the model, 15.9% scored at the ceiling, and when adding the cognition item as a 7th item, 10.9% scored at the ceiling (Fig. 1).

## Discussion

This paper has provided the first evidence for psychometric properties of the breathing and cognition bolt-ons in patients after COVID-19. Such evidence is needed to move bolt-ons from the beta- to approved versions. The findings support the data quality and construct validity of the breathing bolt-on item, and the properties of this item were comparable to that of the commonly used mMRC dyspnea scale. Both had similar score distributions, which is evidence that breathing problems can be assessed using the EQ-5D-5L descriptive system including item wording and response categories. The breathing bolt-on added explanatory power to the five EQ-5D-5L items in regression models using EQ VAS as the dependent variable, an overall measure of HRQoL.The results were more mixed for the cognition bolt-on, including the lack of additional variation explained in the regression models with the EQ VAS as dependent variable. Comparisons of score distributions for the cognition bolt-on are less straightforward due to the CFQ-11 and DSQ-SF having multiple items and different response categories. However, there was evidence for similar response distributions to the most comparable items in the form of DSQ-SF frequency and severity items. There were moderate to high correlations with item scores from the DSQ-SF and CFQ-11, which assess different aspects of cognition. The correlations with DSQ-SF and CFQ-11 scores were considerably higher than those for the EQ-5D-5L dimension scores.

The five EQ-5D-5L dimensions explained around half the variation in EQ VAS scores, the breathing bolt-on making a small and the cognition bolt-on, no additional contribution. The original five EQ-5D dimensions were selected as the most important descriptions of health and hence, additional dimensions might not be expected to make large additional contributions. The EQ VAS is also a single item measure with limitations as criterion measure of general health in this context. Given their higher levels of reliability, summary measures of global, physical, and mental health based on multi-item scales are arguably more appropriate for this purpose. In a recent bolt-on study, EQ-5D-5L dimension scores explained a greater proportion of variation in PROMIS Global Health than EQ VAS scores [43].

Both bolt-ons made small additional contributions to explaining EQ VAS scores in previous studies that used the 3L [16, 44] and 5L versions [17, 43]. In patients with coeliac disease, cognition was found to make the largest additional contribution from five bolt-ons but had a more marginal role in general [45]. In line with our findings, the addition of the bolt-ons slightly reduced the ceiling effect compared to the five EQ-5D dimensions [16, 43, 45]. The numbers reporting no problems for the two bolt-ons compares favorably with those for the study that used the 5L version, both in general and for a population with respiratory disease [43]. For the latter 34 and 71% scored at the ceiling for the breathing problems and cognition bolt-ons, compared to 54 and 40% in the current study.

In another study that tested the 5L version of the breathing problems bolt-on in patients with COPD [13], over 83% reported breathing problems compared to 46% in the current study. Such a difference is expected given the nature of COPD. As in the current study, the majority of breathing problems reported were slight or moderate with very few having extreme problems. Testing for construct validity included comparisons with similar variables reported here including FEV_1_ and the MRC dyspnea scale, with the finding that the inclusion of the breathing problems bolt-on contributed to associations with these clinically important variables [13].

### Strengths and weaknesses

The EQ-5D-5L has had widespread use in studies relating to COVID-19 including hospitalized and non-hospitalized patients, and effects of the pandemic on the general population [46–49]. This is the first study to assess the inclusion of bolt-ons in a fairly representative sample of non-hospitalized patients, and where dimensions of health not included in the original descriptive system are known to be important. The sample size is comparable to several previous bolt-on studies [2, 43] and meant that it was possible to undertake analyses widely undertaken in previous studies of EQ-5D bolt-ons, including following recommended criteria for reporting including PROMs more generally [2, 41, 50]. Hence, consideration was given to data quality, construct validity by means of hypothesis testing, and the relative contribution of bolt-ons to explaining variation in EQ VAS scores.

This community-based study considered broader aspects of health and quality of life relevant to non-hospitalized COVID-19 patients, and hence a range of self-completed instruments were administered concurrently [28, 31].These contributed important information to validity testing, including widely used clinical questionnaires such as the mMRC. Moreover, comparisons were also possible with standardized objective tests undertaken routinely with these patients albeit at different time points. In spite of this, the levels of correlation between the objective measurements and breathing problems bolt-on scores were comparable to those for the mMRC. Correlations between the objective measurements and the cognition bolt-on were similar or higher than those for the EQ-5D dimension scores.

The community-based focus meant that relatively fewer respondents had severe breathing problems compared to those hospitalized with COVID-19 or COPD, or cognitive problems compared to patients with neurological problems. For example, 8% reported an mMRC ≥ 2, which is comparable to the general population (4). Previous bolt-on studies found that patients with COPD experienced more breathing problems [13], and in patients with traumatic brain injury, inclusion of the cognition bolt-on increased the variation explained in EQ VAS scores [44].

## Conclusion

This study provides further evidence for the measurement performance of EQ-5D-5L breathing problems and cognition bolt-on dimensions based on a sample of non-hospitalized respondents with COVID-19 during the first wave of the pandemic in Norway. The results of comparisons of scores distributions, correlations with other instrument scores, and reduced ceiling effects supports further application alongside EQ-5D-5L in populations where such bolt-on dimensions are of potential relevance.

## Appendix 1

Breathing dimension item (bolt-on):

**Table:**

ENGLISH	SWEDISH	NORWEGIAN
Breathing	Andning	Pusting
I have no problems breathing	Jag har inga problem med andningen	Jeg har ingen problemer med pusten
I have slight problems breathing	Jag har lindriga problem med andningen	Jeg har litt problemer med pusten
I have moderate problems breathing	Jag har måttliga problem med andningen	Jeg har middels store problemer med pusten
I have severe problems breathing	Jag har svåra problem med andningen	Jag har store problemer med pusten
I have extreme problems breathing	Jag har extrema problem med andningen	Jeg har ekstreme problemer med pusten

© EuroQol Research Foundation. EQ-5DTM is a trade mark of the EuroQol Research Foundation. This is a modified EQ-5D. Reproduced by permission of EuroQol Research Foundation. Reproduction of this version is not allowed. For reproduction, use or modification of the EQ-5D (any version), please register your study by using the online EQ registration page: www.euroqol.org

## Appendix 2

The following cognitive dimension bolt-on was used in the survey:

**Table:**

ENGLISH	SWEDISH	NORWEGIAN
Cognition (memory, comprehension, concentration, thinking)	Kognition (minne, förståelse, koncentration, tänkande)	Kognisjon *(hukommelse, forståelse, konsentrasjon, tenkning)*
I have no problems with cognition	Jag har inga problem med kognition	Jeg har ingen problemer med kognisjon
I have slight problems with cognition	Jag har lindriga problem med kognition	Jeg har litt problemer med kognisjon
I have moderate problems with cognition	Jag har måttliga problem med kognition	Jeg har middels store problemer med kognisjon
I have severe problems with cognition	Jag har svåra problem med kognition	Jeg har store problemer med kognisjon
I have extreme problems with cognition	Jag har extrema problem med kognition	Jeg har ekstreme problemer med kognisjon

© EuroQol Research Foundation. EQ-5DTM is a trade mark of the EuroQol Research Foundation. This is a modified EQ-5D. Reproduced by permission of EuroQol Research Foundation. Reproduction of this version is not allowed. For reproduction, use or modification of the EQ-5D (any version), please register your study by using the online EQ registration page: www.euroqol.org
