# Correction: Validity of EQ-5D-5L breathing and cognition bolt-ons in non-hospitalized patients after COVID-19

**DOI:** 10.1007/s11136-026-04188-x

**Published:** 2026-02-25

**Authors:** Knut Stavem, Andrew M. Garratt

**Affiliations:** 1 Health Services Research Unit, Akershus University Hospital, Lørenskog, Norway; 2 Institute of Clinical Medicine, University of Oslo, Campus Ahus, Lørenskog, Norway; 3 Department of Pulmonary Medicine, Medical Division, Akershus University Hospital, Lørenskog, Norway; 4 Division for Health Services, Norwegian Institute of Public Health, Oslo, Norway

**Correction to: Quality of Life Research (2025) 35:31**

 10.1007/s11136-025-04133-4

In this article, the footnote to Appendix tables 1 and 2 are missed out. The correct version of tables is provided in this correction.

## Appendix 1

Breathing dimension item (bolt-on):

**Table:** 

English	Swedish	Norwegian
Breathing	Andning	Pusting
I have no problems breathing	Jag har inga problem med andningen	Jeg har ingen problemer med pusten
I have slight problems breathing	Jag har lindriga problem med andningen	Jeg har litt problemer med pusten
I have moderate problems breathing	Jag har måttliga problem med andningen	Jeg har middels store problemer med pusten
I have severe problems breathing	Jag har svåra problem med andningen	Jag har store problemer med pusten
I have extreme problems breathing	Jag har extrema problem med andningen	Jeg har ekstreme problemer med pusten

© EuroQol Research Foundation. EQ-5D™ is a trade mark of the EuroQol Research Foundation. This is a modified EQ-5D. Reproduced by permission of EuroQol Research Foundation. Reproduction of this version is not allowed. For reproduction, use or modification of the EQ-5D (any version), please register your study by using the online EQ registration page: www.euroqol.org.

## Appendix 2

The following cognitive dimension bolt-on was used in the survey:

**Table:** 

English	Swedish	Norwegian
Cognition (memory, comprehension, concentration, thinking)	Kognition (minne, förståelse, koncentration, tänkande)	Kognisjon *(hukommelse*,* forståelse*,* konsentrasjon*,* tenkning)*
I have no problems with cognition	Jag har inga problem med kognition	Jeg har ingen problemer med kognisjon
I have slight problems with cognition	Jag har lindriga problem med kognition	Jeg har litt problemer med kognisjon
I have moderate problems with cognition	Jag har måttliga problem med kognition	Jeg har middels store problemer med kognisjon
I have severe problems with cognition	Jag har svåra problem med kognition	Jeg har store problemer med kognisjon
I have extreme problems with cognition	Jag har extrema problem med kognition	Jeg har ekstreme problemer med kognisjon

© EuroQol Research Foundation. EQ-5D™ is a trade mark of the EuroQol Research Foundation. This is a modified EQ-5D. Reproduced by permission of EuroQol Research Foundation. Reproduction of this version is not allowed. For reproduction, use or modification of the EQ-5D (any version), please register your study by using the online EQ registration page: www.euroqol.org.

The original article has been corrected.

